# A novel spatiotemporal model with advanced feature extraction and unified brain network for depression detection using electroencephalogram signals

**DOI:** 10.1098/rsos.242039

**Published:** 2025-09-17

**Authors:** O. A. Oyinlola, K. A. Gbolagade, I. O. Lasisi, A. W. Asaju- Gbolagade

**Affiliations:** ^1^ Kwara State University, Malete, Nigeria; ^2^ Crescent University, Abeokuta, Ogun, Nigeria; ^3^ University of Ilorin, Ilorin, Kwara, Nigeria

**Keywords:** graph-based major depressive disorder detection, electroencephalogram and spatiotemporal electroencephalogram features extraction, graph convolutional network, gated recurrent unit, k-nearest neighbour, channel–channel relation capturing

## Abstract

Identifying depression using electroencephalogram (EEG) data is a formidable challenge because of the intricacy of cerebral networks and substantial individual variability in neural activity. Conventional models often fail to (i) include the EEG brain connectivity beyond simple paired interactions, (ii) account for brain inter-channel spatial relationships and (iii) integrate a variety of EEG-related features. Addressing these shortcomings, this article presents a novel model, a unified brain network that captures multiple spatiotemporal features that leverage a K-Nearest Neighbour (KNN)-based channel–channel relational matrix and Graph Convolution Gate Recurrent Unit (GCGRU) for depression detection and classification from EEG data, combining Graph Convolutional Networks with Gated Recurrent Units to process both spatial and temporal features of EEG signals. Experimental results demonstrate that the proposed model achieves significant accuracy of 83.67% in major depression disorder (MDD) detection and, with the F1-score, recall and precision reaching 84, 84 and 84%, respectively. Compared with the existing state-of-the-art models for depression detection using EEG, the proposed model achieves 8% improvement in the accuracy of major depressive disorder (MDD) detection.

## 1. Introduction

Depression is a common mental illness that affects a large number of people and is associated with significant suffering as well as massive monetary losses, often surpassing one trillion dollars per year [1,2]. Anxieties are on the rise as a result of people’s jobs and personal problems, and the prevalence of depression has worsened significantly as a result of society’s rapid development. Despite the fact that early identification and treatment may greatly reduce the suffering of depressed individuals, depression diagnosis is still challenging. Due to its subjective nature, the present method of diagnosing depression is prone to errors [2]. This problem has lately seen heavy use of deep learning (DL) and machine learning (ML) models. Analysis of vocal inflections, facial emotions and body language has dominated the literature. But like emotions and words, they may be easily manipulated subjectively and made up. When compared with subjective self-evaluation, electroencephalogram (EEG) testing is a more reliable and valid objective tool for identifying depressive symptoms [3]. According to studies conducted in the fields of psychology and cognitive science, EEG may be used to quantify mental activity. Alpha EEG activity is increased in the resting state of sad persons, according to EEG research across many frequency bands [4]. Additionally, alpha EEG characteristics are higher in subjects with severe depression compared with healthy controls [4].

**Figure 1.**
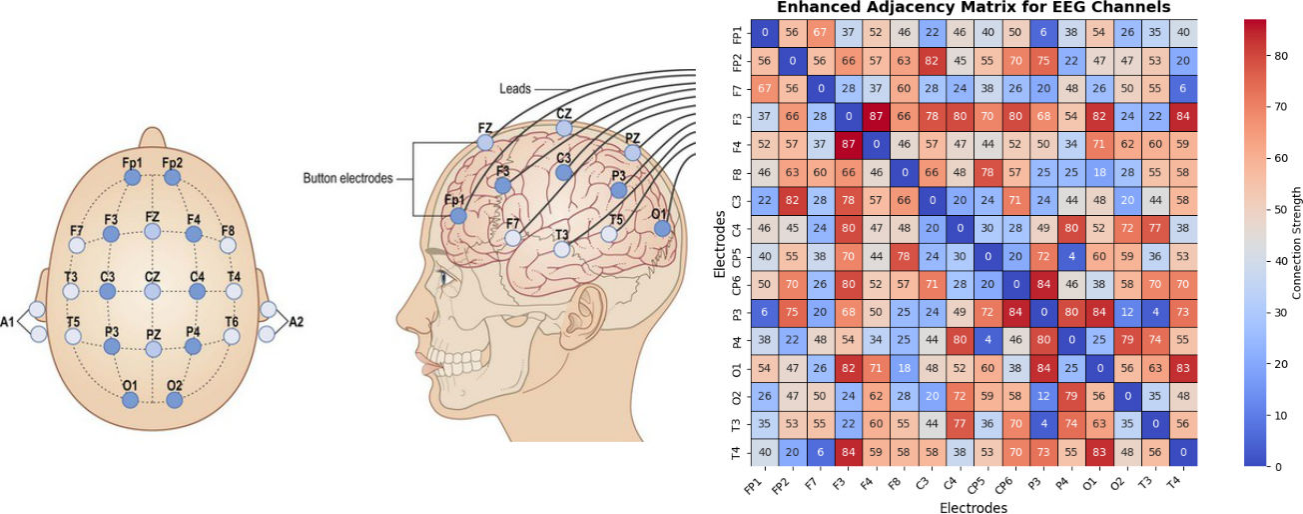
Illustration of electrode position on the scalp and adjacency matrix between the channels [5].

The beta and gamma sub-channel properties of the EEG signals are enhanced, according to research. In order to capture the electrical activity generated by neurons in the brain, EEG electrodes are applied to the scalp, as seen in figure 1. In figure 1, we can see that these electrodes arrange themselves into 16 channels, with each channel representing an individual electrical signal recorded by an individual electrode [6–9]. The signals captured by each channel reflect the spatial activity of several brain areas; these regions differ in terms of both function and connection, and they also change over time. Therefore, in order to conduct EEG-based depression categorization in this study, the aforementioned results provide a solid theoretical foundation [8].

### 1.1. Related work

The recent EEG-based studies are categorized as ML and DL. The studies based on ML include the extraction of manually produced channel features of EEG, as shown in figure 1, or diverse statistical characteristics, including spectrum analysis, alpha asymmetry, brain laterality, signal-based features and network-based features [10]. The second approach is to depend on DL, which may be categorized as Convolutional Neural Networks (CNNs) [8] and Long Short-Term Memory (LSTM) [8], CNN-LSTM [11] and Deep Hybrid Neural Network (DeepHNN: CNN-LSTM) [12]. Furthermore, Graph Convolutional Networks (GCN) has been a significant technique in recent years [1,7,13].

Several experts have asserted that the topological nature of EEG data warrants consideration. They employed GCN and presented several preconfigured graphs or adjacency matrices for the identification of depression. The authors in [7] computed EEG’s differential entropy characteristics to formulate the predefined graph’s adjacency matrix utilizing its Pearson matrix. In [14], the authors explore self-attention to consider the local and global channel-wise importance within the same data, ignoring the inter-layer connections. The authors in [15] employ power spectral density to generate the node features and employ phase locking value to construct the pre-defined graph. The model in [16] constructs the adjacency matrix based on Euclidean distance between the channels, ignoring the strength of each channel. Due to the dynamic nature of brain networks, a complete topological structure that captures both spatial and functional links between EEG channels is needed to better recognize inter-channel interactions. Adapting the adjacency matrix might improve feature aggregation and depression diagnosis.

Several studies have highlighted the potential of spatial–temporal analysis in EEG-based depression identification. Models in [8,11,12] consider CNN for spatial feature extraction from EEG signals and are further explored through temporal models like LSTM for depression detection. The power spectra of Theta, Alpha and Beta frequencies were extracted and projected onto the brainpower spectrum to determine electrode positions [17]. After superimposing and classifying three brain maps, CNN and Gated Recurrent Unit (GRU) were used to extract and classify sequence characteristics in [17]. The CNN-based algorithms interpret EEG data as pictures and develop diverse convolutional kernels to extract features, although they do not fully account for the interrelationship across multiple channels. Nonetheless, these methodologies continue to extract temporal and spatial data independently, neglecting to account for the inter-channel connections at various temporal intervals. While EEG studies have shown promising outcomes in many activities, they cannot be immediately applied to MDD detection tests. The challenge of integrating spatial structure and temporal dependency information in depression patients' varied brain networks remains the focus of the article.

In graph-based EEG spatiotemporal algorithms, EEG channels are typically represented as nodes in a graph. As shown in figure 1, these nodes (represented by circles) and the connections between them are stored as an adjacency matrix. Models like those in [7,13,14] adopt graph-based methods such as Self-attention Graph Neural Networks (GNN) and GCN. Here convolution operations are applied to aggregate nodes and generate meaningful embeddings for depression classification [7]. GNN-based methods utilize pre-computed graph adjacency matrices to depict the interrelations across different EEG channels but fail to capture the dynamic variations for each EEG brain network. That is, brain networks are not only different for depressed and healthy individuals but also change over time. Thus, existing algorithms still face the following challenges:

— Existing models like in [7,13,14] construct the adjacency matrix based on the Euclidean distance between the channels. In reality, certain channels have stronger correlations or interactions with specific others, especially in the context of cognitive functions like depression. Existing methods ignore the inter-channel spatial relations as they play a critical role in aggregating features from other channels.
— In EEG-based research, especially in mental health diagnosis (e.g. depression), the focus is on analysing how brain signals vary both across time and between subjects. In existing algorithms like in [14], EEG data are represented for each subject using a three-dimensional tensor, ignoring the time evolution of EEG signals across each subject. Traditional methods often rely on simplified representations that fail to account for the variations in EEG brain connectivity across subjects.

### 1.2. Motivation and unique contribution of the article

The present study is motivated to present a unified model that allows for the inclusion of population-wide characteristics, which can be leveraged to identify common EEG variations across subjects while maintaining individual differences. Compared with the previous models [7,13–17]: (i) For each subject, the EEG data are represented as a four-dimensional tensor where the time evolution of EEG signals across multiple subjects is incorporated. (ii) The adjacency matrix is generated by considering the spatial distance between channels and incorporating the most relevant connections (sparse representations). This creates a more realistic graph structure that reflects how electrodes (and the underlying brain regions) are spatially and functionally related. (iii) The spatial and temporal features are fused as a population network where for each subject, the adjacency matrix, tensor and respective label are stored for depression detection and (iv) Existing methods where convolution pooling is used for node aggregation, the proposed model employs a max pooling method that captures the essential channels from the adjacency matrix and learned spatiotemporal features based on scores allotted. Thus, the main contributions of the article are:

— The article introduces a novel advanced DL pipeline termed multi-segment spatiotemporal graph recurrent unit with graph pooling, significantly enhancing detection accuracy by retaining the essential channels. It unveils a cohesive framework to decipher the intricate connections between channel–channel relation and segment-wise fusion of spatiotemporal features via GC-GRU (Graph Convolution Gated Recurrent unit).
— The proposed method captures channel-wise features over time for each segment and stores them in a four-dimensional tensor with subject ID, channels, features of the channel and time as segment-wise spatiotemporal features. This allows the model to simultaneously account for both the temporal and spatial dynamics, integrating both aspects effectively. The article introduces an enhanced adjacency matrix to store the channel–channel relations that capture the spatial distances of EEG channels and incorporate the most relevant connections between channels using KNN (K-Nearest Neighbour). It is dynamically calculated for each subject, considering individual brain networks.
— The article proposes to fuse the corresponding segment-wise spatiotemporal feature and channel–channel relations as a unified network. This is stored for each channel segment as ID, adjacency matrix, tensor and label (depression or normal). The unified brain network is trained over max-pooling-based GC-GRU to generate spatiotemporal feature embeddings for depression detection. This way the proposed model ensures that channels play a significant role in highlighting the EEG variation causing depression. This allows the model to differentiate between important and less essential nodes (channels) in a unified brain network for depression classification.

Liu *et al*. [17] managed the spatial and temporal characteristics of EEG signals for the identification of depression, which is the key difference between their work and the proposed work. Liu *et al*. [17] plot electrode sites using orthogonal projection and superimpose Theta, Alpha and Beta brain maps in order to obtain spatial and frequency-domain information. After that, it uses a straightforward CNN-GRU model with six layers for classification, which achieves good accuracy at little complexity. By creating a channel–channel relational matrix based on KNN to capture spatial connection across EEG channels, the suggested approach, on the other hand, creates a more complex brain network model. It integrates GRU and GCN to understand temporal dynamics and spatial relations in depth. The suggested study innovates by combining graph-based spatial modelling with sequential learning, providing substantial progress in capturing complex EEG interactions, whereas Liu *et al*. [17] employ conventional convolutional techniques for feature extraction.

The article is organized into sections: §2 explains the proposed methodology’s details. In §3, the results and discussions are conducted, and the conclusions are drawn in §4.

## 2. Preliminaries

Prior to examining the workings of the proposed model, the preprocessing of EEG data, the formation of a four-dimensional subject tensor and the operation of the GC-GRU will be addressed first. This establishes the foundation for understanding the model’s functionality.

### 2.1. Electroencephalogram data preprocessing and preparation

Distinct brain areas exhibit varying levels of activity and engage in interactions with one another. Viewing these brain areas as channels, EEG brain activity is not confined to a single channel but involves interaction among numerous channels. Furthermore, EEG signals fluctuate across time due to the continuous changing of stimuli of the bodily and the interaction among brain channels. Examining the interactions across these channels and the temporal variations of these signals is crucial for recognizing patterns linked to mental health issues, such as depression, and for comprehending functional connections within the brain. Diagnosing depression with existing algorithms often requires the pair-wise based and pre-computed adjacency matrix, which fails to account for the variations in brain network connection across depressed individuals with good health EEG records channel-wise data from each subject, capturing signals over a period. But this raw data are not readily used for analysis of spatiotemporal features. Thus, necessary pre-processing is applied. The EEG data are processed subject-wise and for every subject, EEG signal, channel–channel relations and Beck Depression Inventory (BDI) score are processed.

*Dataset description:* The EEG dataset used in this study was originally collected by researchers at the University of New Mexico and Arizona State University and is publicly available via OpenNeuro (https://openneuro.org/datasets/ds003478/versions/1.1.0) [18]. The dataset includes resting-state EEG recordings and behavioural assessments from participants recruited through introductory psychology courses based on their scores on the BDI. The BDI is a widely used, standardized self-report instrument designed to assess the severity of depressive symptoms [18]. Scores range from 0 to 63, with higher scores indicating greater levels of depression. According to [18], a total of 122 participants were assessed, of whom 46 exhibited elevated depressive symptoms (BDI ≥ 13) over multiple testing intervals and were categorized into the depressed (DEP) group. The remaining participants formed the control (CTL) group (BDI < 7), with no reported history of major depressive disorder (MDD) or other Axis I psychiatric conditions, as verified by self-reports and the Electronic Mini International Neuropsychological Interview [18]. EEG signal data were acquired using a 66-channel Ag/AgCl electrode cap, with bandpass filtering from 0.5Hz to 100Hz and a sampling rate of 500Hz. Electrode impedance was kept below 10 kΩ throughout the recording [16]. In this study, a subset of 42 participants (22 highly depressed, 20 completely normal) was selected for model training and evaluation. These participants were chosen to ensure a clear contrast between groups, with stable BDI scores over time, minimal comorbidities and high-quality EEG recordings. While the BDI provides a continuous measure of depressive symptom severity, we adopted a binary classification framework (MDD versus CN) to align with prior works [19–27] and to simplify the model experimentation and state-of-the-art models.

The proposed model uses data collected from 42 individuals, 20 of whom had HC and 22 of whom had MDD, drawn from an open Neuro database. The three main data file sources that are used are *electrodes.tsv*, *participants.csv* and EEG data files (*run−01* and *run−02*). The purpose of these files is

(1) Data sources*—run−01* and *run−02* denote the files corresponding to each subject’s test run 1 and test run 2, respectively. Only the *run−02* data is selected for the analysis due to better quality and less noise, ensuring more accurate results.
(2) Electrode coordinates—Each subject has a unique *electrodes.tsv* file, which records the positions of the electrodes on the scalp during EEG testing.
(3) EEG signal data—EEG signal files stored in .*set* files. The .*set* files of EEG signal are read and processed by a particular python package minimum norm estimation.

After loading the files, the pre-processing procedure begins. The processing is conducted subject-wise. In the first stage, for each subject in $S={\{S}_{1}\ldots S_{42}\}$, the EEG signal is processed channel-wise. Here for each channel in $C=\left\{C_{1}\ldots C_{16}\right\}\in R^{16\times 3}$, the raw EEG signals are for 16 channels over time 184.093 s. In this article, the 16 channels’ data, i.e. Fp1, Fp2, F3, F4, C3, C4, P3, P4, O1, O2, F7, F8, T3, T4, T5 and T6 are used. For each channel in C, the signal is processed for each timestep t. For each step in τ={1,2,…,184093} , the EEG signal Sig is recorded as a matrix in $R^{C\times \tau }$ representing channel readings over time. Then the mean value of the signal for each channel over the entire period is calculated as given in step 1 .a.(iii) in algorithm 1.

After channel-wise processing of EEG signal for one subject is complete, a band-pass filter and a notch filter are used to eliminate noise and artefacts from the EEG data. The band-pass filter retains signals within a designated frequency band, while the notch filter removes line noise. Independent component analysis (ICA) is then used to further separate and eliminate noise from the EEG signal. The clean signal data are then resampled to a new frequency of 500 Hz. This is accomplished by first downsampling the data by a factor of 200 to ensure uniformity across various signals, yielding a downsampled frequency of 200 Hz and an effective sampling frequency of 500 Hz. Each resampled EEG signal is segmented in $seg=\{seg_{1}\cdot \cdot \cdot seg_{490}\}$ using a window size of 6000 data points, with a 3000 point overlap between subsequent windows to capture temporal dynamics over time. Thus, this process is considered for each subject and multi-segment data is captured, which are combined and given as $seg={\left(16\times 73600\right)}_{1}={\left(16\times 6000\right)}_{1},{\left(16\times 6000\right)}_{2}\ldots ..{\left(16\times 6000\right)}_{490}$. This way, spatiotemporal brain activity across different subjects is also considered. Given that the time of each subject’s data may differ, the segmentation procedure is changed appropriately, resulting in a distinct set of segments for each subject. For each segment, compared with previous work like [14,16], where only alpha and beta features are captured, in this article, eight signal features are captured. For each seg of the EEG signal data, a range of features is computed to capture various characteristics of the brain signals:

— *Mean*: The average value across each segment is computed.
— *Lempel-Ziv Complexity (LZC)*: A measure of the complexity of the signal, capturing its compressibility.
— *Higuchi Fractal Dimension (HFD)*: A measure of the fractal dimension of the EEG signals, providing insights into their self-similar structure.
— *Detrended Fluctuation Analysis (DFA)*: A method for assessing the long-term correlations within the EEG signals.
— *Frequency Bands*: Power values in key EEG frequency bands (Delta, Beta, Gamma and Theta) are calculated to represent various brain states.

For each segment of signal in seg the EEG signal features of shape $\left(C\times f\right)$, that is 16 channels and 8 and features a two-dimensional matrix, is captured. Finally, this information is stored as a four-dimensional tensor and segment-wise spatiotemporal features are given as $Tensor=\left\{C\in \left[1,16\right],f\in \left[1,8\right],seg\in \left[1,490\right],\tau \in \left[1,184093\right]\right\}$.

**Figure:**
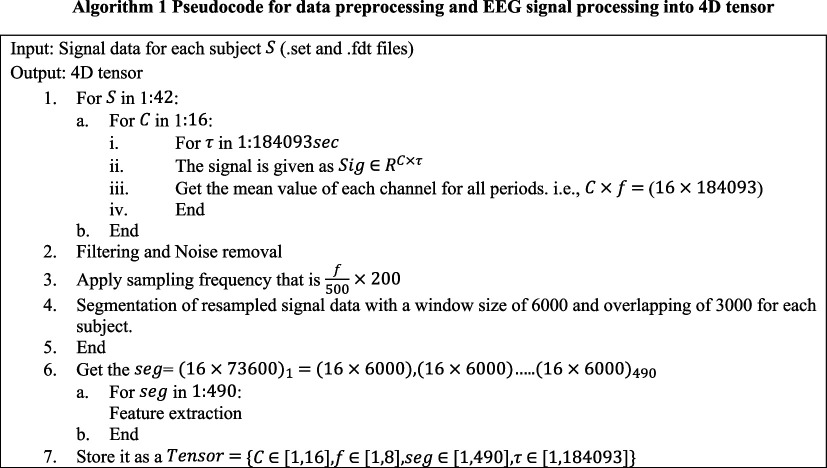


Once the four-dimensional tensor is obtained, the channel–channel relation matrix is then calculated. EEG signal is recorded by placing electrodes on the scalp to track brain activity; each electrode is given a unique three-dimensional spatial coordinate (X, Y, Z) that represents its exact location on the scalp. Electrode coordinates are grouped in a matrix. Prior research, for example in [14] and [16], has computed the pairwise Euclidean distances between each electrode in order to assess the connections between various channels. Adjacency matrices, that are based on the distance from two points in space, connect all channels of the brain. This may introduce irrelevant connections that are far away, which can reduce the significance of localized brain networks. As a result, the KNN technique is used to more accurately record significant inter-channel interactions. By limiting channel connections to those immediately around them, this method improves the realism of depictions of localized brain interactions. The channel–channel relations are extracted using the KNN algorithm, which selects the closest k channels for each channel in $C\in R^{16\times 3}$ (16 channels, each with three-dimensional coordinates), based on the distances calculated (Euclidean distance) and define an edge between them. These channel-to-channel relations (edges) that are within a predefined distance threshold (thr) are considered to be connected. The adjacency matrix $A_{S}$ stores this information. Algorithm 3 lays forth the specific procedures.

**Figure:**
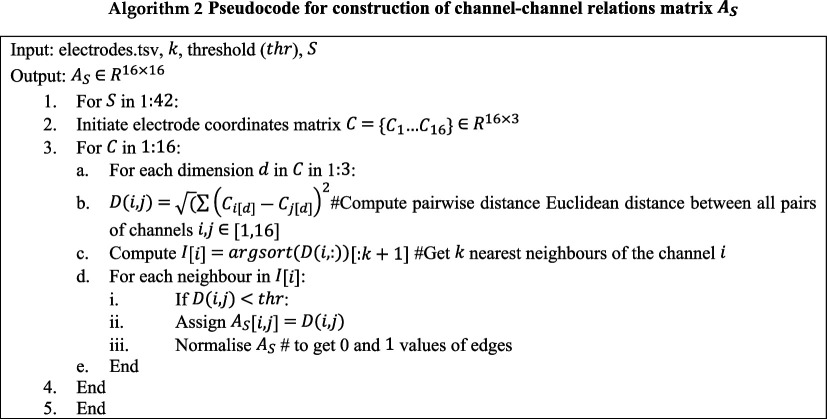


### 2.2. Graph convolution gate recurrent unit working

In this study, a graph convolution gate recurrent unit (GCGRU) module is employed, which combines the GCN structure and the GRU structure to perform spatiotemporal feature extraction on the unified brain network. The preliminary working of these modules is as follows:

#### 2.2.1. Graph convolutional networks module

A graph G is represented by a set of nodes V (vertices) and edges E, denoted as G=(V,E). In GCNs, each node has associated features. A GCN aggregates the features of each node’s neighbours to compute a new representation for each node. For a single GCN layer, the propagation rule is defined as:


(2.1) $$f_{l}=ReLU(D^{\frac{-1}{2}}A_{S}D^{\frac{-1}{2}}X_{S}^{t}W_{l})),$$

where $f_{l}$ is the node features at layer l, $A_{S}$ is the channel–channel relation adjacency matrix, D is the degree matrix of $A_{S}$, $W_{l}$ is the learnable weight at layer l and $X_{S}^{t}$ is the feature matrix. $D^{\frac{-1}{2}}A_{S}D^{\frac{-1}{2}}$ operation normalizes the adjacency matrix before operations as per [7].

#### 2.2.2. Gated recurrent unit module

The GRU module captures the temporal dependencies in the EEG signal. They are a simplified version of LSTM networks but with fewer parameters, which result in faster training. A GRU cell has two principal gates—the update gate and the reset gate—that govern the information flow inside the cell.

For each time step t, the GRU cell receives an input vector $F_{S}^{t}$ from the GCN embeddings and a hidden state from the previous time step $h^{t-1}$. The cell computes an updated hidden state $h_{t}$ based on $F_{S}^{t}$ and $h^{t-1}$. The reset gate $r^{t}$ determines the extent of prior information to be discarded. When the reset gate approaches 0, it disregards the prior hidden state, concentrating only on the present input. The reset gate is calculated as follows:


(2.2) $$r^{t}=\sigma \left(W_{r}\left[f\left(X_{S}^{t}\right),h^{t-1}\right]+b_{r}\right).$$

The update gate $u_{t}$ regulates the retention of the previous hidden state and the incorporation of new information. It regulates the balance between retaining prior memory and integrating new data. The update gate is calculated as follows:


(2.3) $$u^{t}=\sigma \left(W_{u}\left[f\left(X_{S}^{t}\right),h^{t-1}\right]+b_{u}\right).$$

The GRU calculates a candidate hidden state $c^{t}$, which signifies the new information that may be incorporated into the memory. The candidate hidden state employs the reset gate to regulate the impact of the preceding hidden state $h^{t-1}$. It is computed as


(2.4) $$c^{t}=tanh(W_{c}\left[\;f\left(X_{S}^{t}\right),(r^{t}⊙h^{t-1})\right]+b_{c}).$$

The final hidden state $h^{t}$ is computed by combining the previous hidden state $h^{t-1}$ and the candidate hidden state$c^{t}$ using the update gate $u^{t}$:


(2.5) $$h^{t}=u^{t}⨀h^{t-1}+\left(1-u^{t}\right)⨀c^{t}.$$

From equations (2.2) to (2.5), the bias and weights are given as ($W_{r}$, $W_{u},W_{c}$) and ($b_{r}$, $b_{u},b_{c}$), respectively, and ⨀ denotes element-wise multiplication. This configuration allows the GRU to selectively preserve or modify information, making it particularly effective for sequential data when some previous knowledge is crucial for current predictions.

## 3. Proposed methodology

### 3.1. Overview of the proposed model

**Figure 2.**
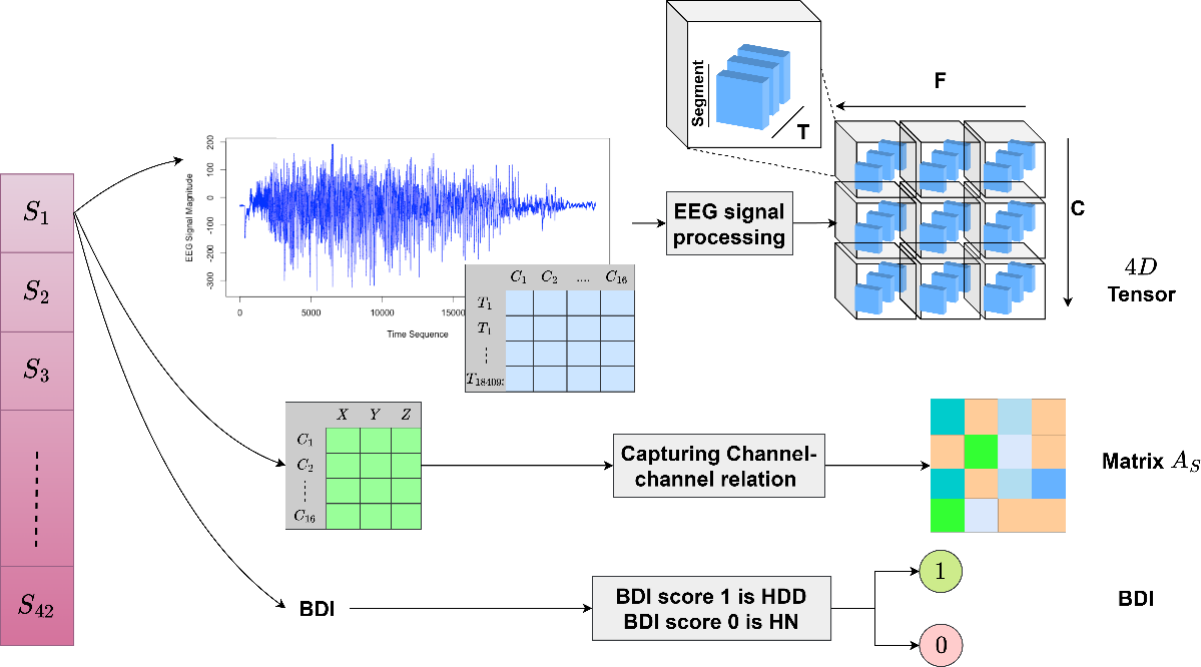
Four-dimensional tensor, $A_{S}$ channel-channel matrix and BDI score extraction.

This article proposes a framework for classifying depression levels (Highly Normal or High Depression Disorder) from EEG signals by leveraging spatiotemporal feature extraction and a GCGRU model. The method focuses on constructing a unified brain network from extracted segment-wise spatiotemporal EEG features. The main stages of the proposed model are: (i) spatiotemporal fusion data preparation, (ii) preparation of unified brain network and pooling-based GCGRU model and (iii) classification model. The overall flow of the model is illustrated in figure 2, the process begins with initializing the EEG signal data. Each subject’s EEG signal is captured across 16 channels. The raw EEG signal undergoes preprocessing and segmentation into smaller time windows. The spatiotemporal fusion data is prepared as a four-dimensional tensor for multiple segments and channel–channel relations. A relationship matrix is computed between channels (denoted as $A_{S}$), which captures the inter-channel dependencies. This is calculated using KNN (K-Nearest Neighbours) based on the distance between channels. This information is used to represent the brain network structure fused as a tuple. This process is repeated for each channel and the Unified Brain Network is constructed as a list of tuples shown in figure 2. The data from each subject is represented as a combination of the tensor, the channel relation matrix and a binary indicator (e.g. 0: Healthy Controls (HC) or 1: Major Depression Disorder (MDD)), this hierarchical fusion results in a Unified Brain Network, capturing channel–channel local relationships at a low level and integrating multi-segment, subject-specific data at a higher level. This is used as input to the GC-GRU model to capture the spatiotemporal embeddings. These embeddings are improved using max-pooling and channel–channel relations for the final vector and classification.

**Figure 3.**
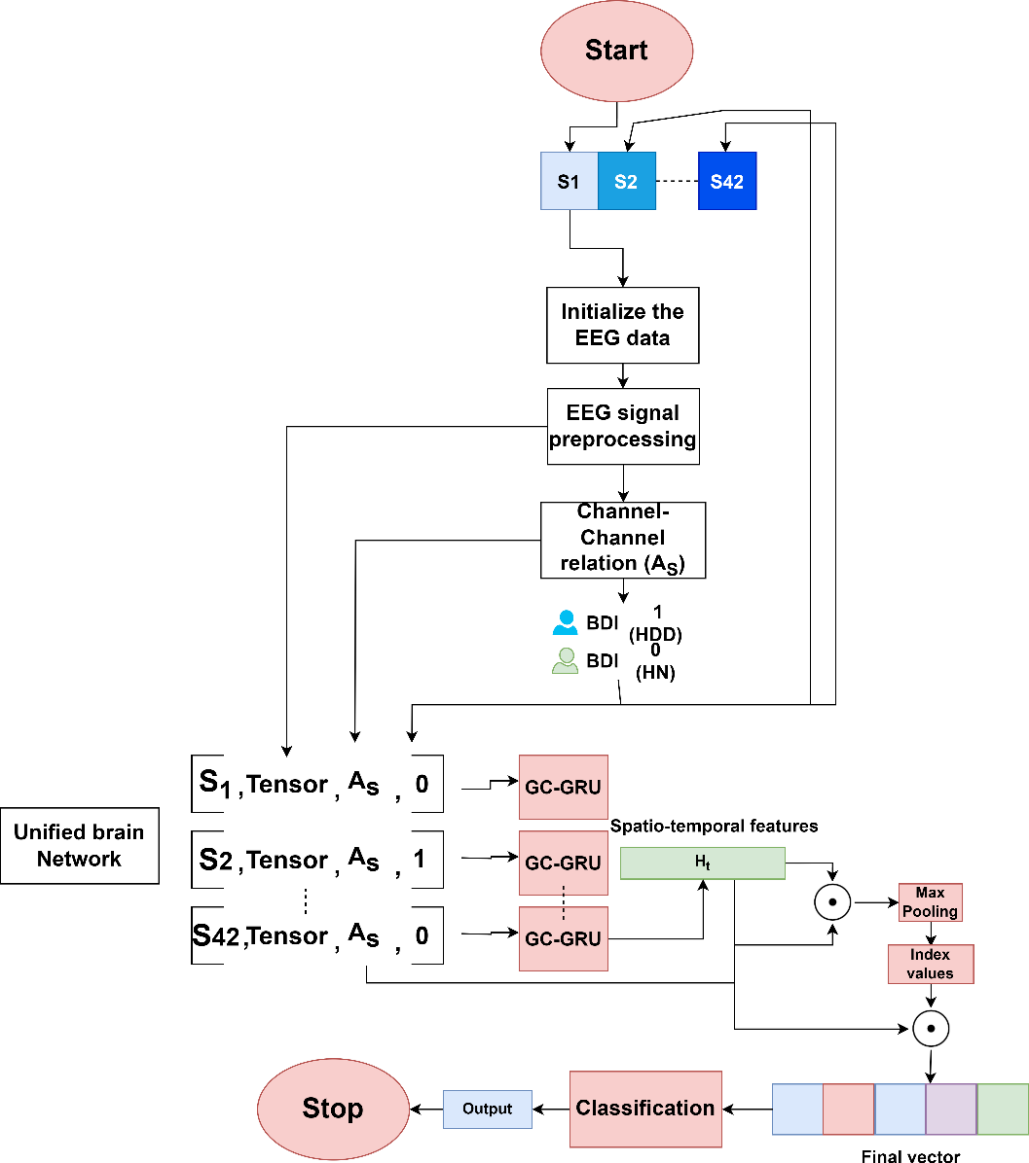
Flow chart of the proposed model for depression detection.

The steps for the process flow, as explained in figure 3, are given as,

(1)* Spatiotemporal fusion data preparation and four-dimensional tensor generation*: The EEG signal data for multiple subjects $S={\{S}_{1}\ldots S_{42}\}$ is processed to extract meaningful features. For each subject $S_{i}$, EEG signals from multiple channels are processed by first calculating the mean signal value across time for each channel. The signal is then filtered using band-pass and notch filters, and noise is removed using independent component analysis (ICA). Afterward, the signal is downsampled to a specific frequency and segmented with a defined window size and overlap. From each segment, various features are extracted, mentioned in §2.2. The extracted features are stored as a four-dimensional tensor for further analysis.
(2) *Preparation of unified brain network and pooling-based GCGRU model*: The unified brain network for each tuple is stored in a list as

(3.1) $$EEG_{unified}=\{S_{1},A_{S_{1},}T_{seg_{1},C,f,t},y_{1},S_{2},A_{S_{2}},T_{seg_{2},C,f,t},y_{2},\ldots ,S_{i},A_{S_{i}},T_{seg_{i},C,f,t},y_{i}\}$$

Here *i* and *j* are the number of subjects, segments and is the corresponding label for the subject as shown in figure 2. The GCGRU model is run for each list in Tuple to generate the final spatiotemporal embeddings $H^{t}$. To retain the important channels, masking and max-pooling are applied, and final vector representations $Final_{\to }$ are considered for classification as given in Steps 5−9 in algorithm 3. This process is detailed in §2.3

(1) *Classification Model*: These vectors are applied to a fully connected layer and Softmax for depression classification.

**Figure:**
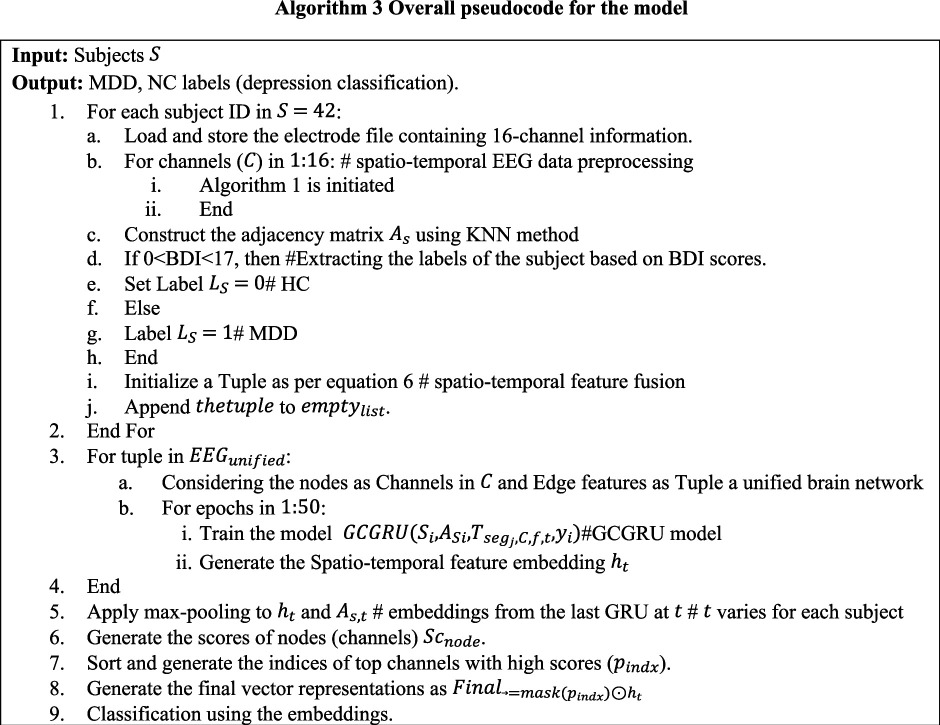


**Figure 4.**
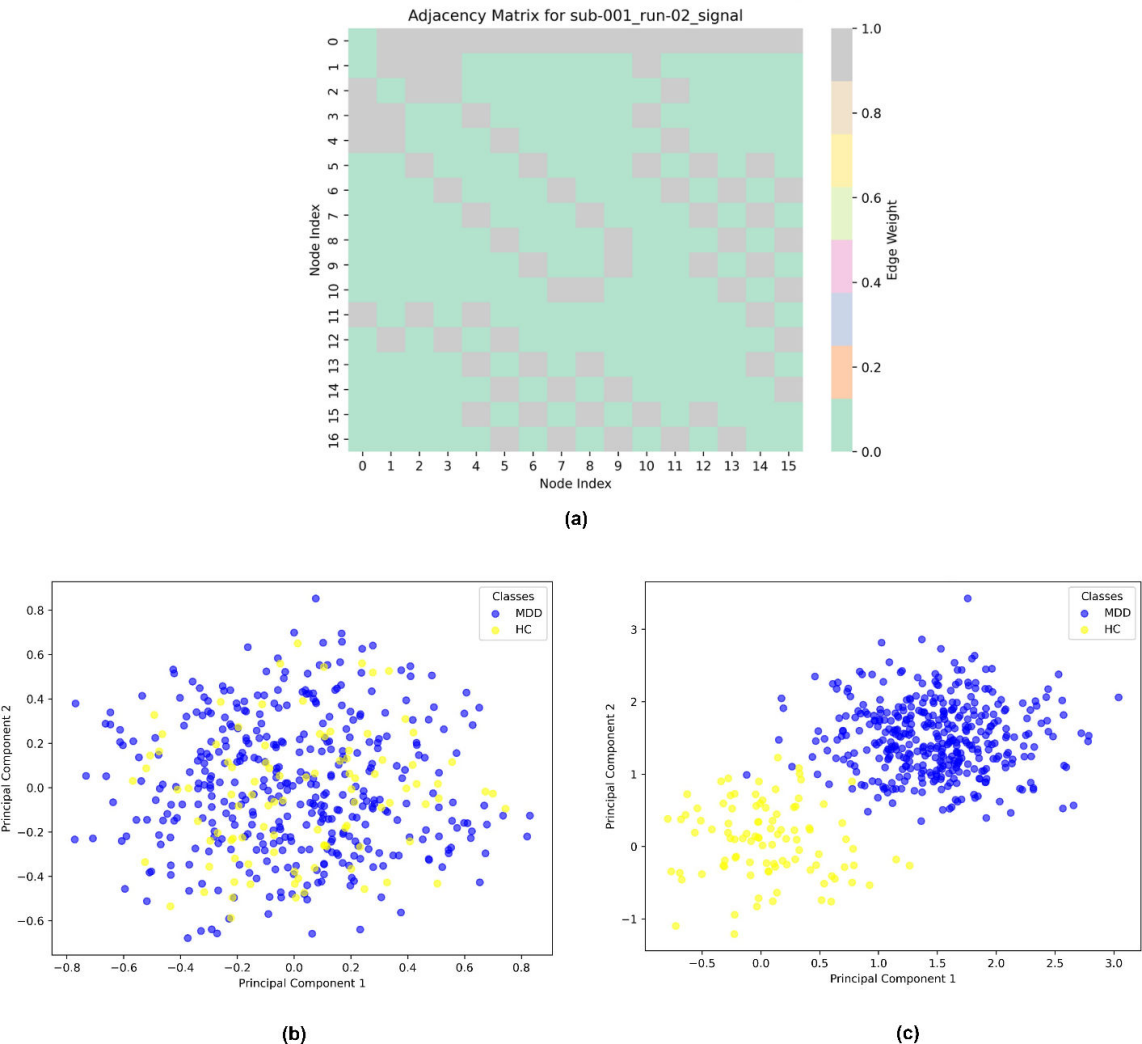
Illustration of channel-channel relations ($A_{S}$) for a subject, clustering the embeddings from the model where (b) $A_{S}$ without KNN method (c) $A_{S}$ with KNN method.

As given in algorithm 3, for each subject and channel information in electrodes.tsv, the KNN-based adjacency matrix is generated. The $A_{S}$ from a single subject is given in figure 4a, where node index represents the 16 channels and edge weights are computed through step 3 (d). As per step d, the adjacency matrix $A_{S}$ only includes edges (connections) for neighbours whose distance is less than the defined threshold. This process creates a sparser matrix, emphasizing connections that are both close in proximity and meaningful in terms of signal interaction. The improvement from this approach is highlighted in the embedding clusters shown in figure 4b,c. The coloured data points represent the depression labels derived from BDI scores, as described in algorithm 1. In figure 4b, the data points for MDD (blue) and HC (yellow) show significant overlap, indicating poor separation between the two classes when using a traditional distance-based adjacency matrix. This overlap occurs because computing the distance between all channel pairs fails to prioritize meaningful local interactions, which are key to differentiating the two conditions. In contrast, in figure 4c, where KNN is applied as described in step 3(c) of the algorithm, the model achieves a much clearer separation between the MDD and HC classes. By focusing on influential neighbouring channels through KNN, the model effectively captures the local channel–channel relationships, which are crucial for distinguishing between the two conditions. This improvement demonstrates how incorporating KNN-based adjacency matrices enhances the model’s ability to differentiate between MDD and HC, leading to better classification performance. The connectivity graph’s richness and sparsity are directly impacted by the K selection in our approach: while a bigger K incorporates broader interactions that may contribute noise but also record more global brain dynamics, a smaller K highlights stronger, more concentrated relationships between EEG channels, possibly maintaining more significant physiological correlations. Based on cross-validation studies, we empirically chose K to guarantee the best balance, finding that moderate values of K offered the optimum trade-off across complexity of the model and performance.

In our approach, the KNN threshold (the number of nearest neighbours, K) is critical in shaping the EEG channel-related graph. A smaller K results in a sparser graph that exposes just the strongest local spatial linkages, perhaps boosting noise robustness while leading to the loss of some worldwide brain network patterns. A higher K, on the other hand, results in a denser graph, which captures more extensive connections but may also introduce redundant or less significant interactions. We chose a K value that optimizes model performance by balancing local and global connections through methodical adjustment.

By preparing the EEG data in this way—through channel-channel relations and multi-segment based four-dimensional tensor—the study captures both the spatial (across brain regions) and temporal (across time) dynamics of brain activity, leading to a comprehensive representation of functional connectivity in the brain. This is explained in the next section.

### 3.2. Construction of unified brain network and capturing spatiotemporal features using the channel-wise pooling-based graph convolution gate recurrent unit model

The construction of the unified brain network serves as the foundation for modelling the complex inter-channel relationships in EEG data for depression classification. Thus, in this section, construction of the unified brain network and the process of extracting meaningful embeddings from channel-wise max-pooling-based GCGRU is detailed.

EEG signals, in their raw state, capture brain activity but do not directly expose how these activities evolve over time or interact across regions. Without structuring the data, the signals appear as a collection of individual snapshots rather than a coherent narrative of brain function. As discussed in §2.2, for each subject the four-dimensional tensor and channel–channel relation is prepared from the EEG data. This is given as $EEG_{unified}$ in Algorithm 1-step 3. For $S={\{S}_{1}\ldots S_{42}\}$, a tuple is formed containing the corresponding adjacency matrix, the four-dimensional tensor and the subject label $y_{i}$, forming the unified representation as given in equation (2.1). Considering the edges as the tuple and channels as nodes, a unified brain network is constructed. This step combines all the subject-level data into a unified EEG graph where channels are represented as nodes, and the relationships between channels are the edges as shown in figure 4. Thus, the brain network is denoted as $G_{S}^{t}=(C,A_{S},Tensor)$ and the unified brain network from equation (2.1) is represented as, $EEG_{unified}=\{\left(S_{1},G_{1}^{t},y_{1}\right),{(S}_{2},G_{2}^{t},y_{2}),\ldots .(S_{42},G_{42}^{t},y_{42})\}$. Specifically, for each subject in S, using $A_{S}$ as edge and Tensor at the time period recorded at t as node features and channels as nodes a unified brain network is constructed ($G_{S}^{t}=\{G_{1}^{t},\ldots .G_{42}^{t}\}$). Finally, for these series of brain networks ($G_{S}^{t}$), multiple GCGRU models are stacked to capture the spatiotemporal features ($h_{t}$). These features are subjected to self-attention graph pooling as in [28] to generate the final vector representations for depression classification. This process is detailed in the next section and illustrated in figure 4.

#### 3.2.1. Graph convolution gate recurrent unit model with channel-wise max-pooling

EEG data vary spatially as well as temporally and existing studies propose CNN-LSTM [12], GCN-based method for EEG classification [7,13–15], but ignore the temporal variations in the EEG data. Whereas, models like in [17,28] propose using the GRU model for extracting spatiotemporal features. The LSTM [12] and the GRU [17] are variants of recurrent neural networks that have proven effective in resolving the previously described challenges. The core ideas of LSTM and GRU are analogous. The algorithm employs a gated system to effectively retain substantial long-term knowledge and is equally adept at executing various tasks. Nonetheless, the LSTM model’s complex design leads to an extended training period, whereas the GRU model features a relatively simple structure, fewer parameters and expedited training efficiency. Consequently, the authors used the GRU model to elucidate the temporal correlations within the brain region data, as seen in figure 4. Thus, in this study the GCGRU module is employed, combining the GCN structure and the GRU structure to perform spatiotemporal feature extraction on the unified brain network as shown in figure 4. The spatial relations are extracted for each subject S with feature $X_{S}^{t}$ using the residual-based graph convolution operation as in [29],


(3.2) $$F\left(X_{S}^{t}\right)=cat(f_{1}\left(X_{S}^{t}\right),f_{2}(X_{S}^{t}))$$

where $f_{1}\left(X_{S}^{t}\right)$and $f_{2}(X_{S}^{t})$ are the output feature maps after applying two layers of graph convolution calculated as per equation (2.1).

Once the spatial features $F\left(X_{S}^{t}\right)$ are extracted for each time step t of each subject in S, the GCGRU cell processes them to capture temporal dynamics. The GCGRU cell is a modification of the standard GRU cell that incorporates graph convolution into the update mechanism. The mathematical equations from 2−5 are used in the GRU cell. $h^{t}=GRU(F\left(X_{S}^{t}\right))$ is the final hidden state output for the GCGRU model. Further, the importance of channels (nodes) is extracted through attention pooling as given in [28], shown in figure 4.

The max-pooling operation is applied to capture the most relevant channel features as shown in figure 2. After processing the sequential graph data, the output from the GRU is aggregated to create a pooled representation. This is given as


(3.3) $$Sc_{node}=ReLU\left(A_{S}^{t}h^{t}W_{pool}+b_{pool}\right).$$

The $A_{S}^{t}h_{t}$ from the last GCGRU in the stack as shown in figure 4 and $W_{pool},b_{pool}$ are weight and bias. The max-pooling is computed to select the indices of highest activations in equation (3.3), given as


(3.4) $$p_{indx}=argmax\left(Sc_{node}\right).$$

Thus, the final vector representation is computed and this is shown as attention-masking in figure 4,


(3.5) $${\text{Final}}_{\to =\sum_{n=1}^{p_{indx}}mask\left(p_{indx}\right)⊙h^{t}}.$$

*Depression classification model:* The final vector representations from equation (3.5) are passed through fully connected layers to generate the depression probability (P(y∨X)), explained as


(3.6) $$P(I)=softmax\left(W_{f}\cdot {\text{Final}}_{\to }+b_{f}\right).$$

It represents the probability of class in $y_{i}$ given input I. The $P\left(I\right)$ is the final predicted output of the classification model. The accuracy of these predictions is evaluated in the subsequent section, where model performance is assessed, and optimization techniques are used to enhance precision.

## 4. Results

In this section, the simulation environment for the model is discussed, then the ablations of the model alongside the state-of-the-art model comparison.

### 4.1. Simulation environment and optimization

The experiments were performed on Google Colab Pro, and the settings involved are given in table 1.

**Table 1.** Simulation parameters.

parameter	value
*GPU*	Nvidia Tesla T4
*GPU VRAM*	15 GB
*RAM*	51 GB
*optimal hidden dimension*	64
*subjects*	42
*EEG segments*	490 (232 MDD, 248 HC)
*batch size (GCGRU)*	40 EEG clips
*epochs (GCGRU)*	100
*dropout probability*	0.7
*loss function (GCGRU)*	Binary Cross-Entropy with Logits

EEG data collected from 42 subjects were preprocessed into 490 segments, with 232 segments for the MDD class and 248 for the HC class. The GCGRU model is trained with a batch size of 40, using 100 epochs, a dropout probability of 0.7 and binary cross-entropy with logits loss.

To ensure clarity for both ML practitioners and psychological scientists, we provide additional details on how our dataset (*n* = 42) was used in training and evaluation. The article proposes a segmentation that yielded a total of 490 EEG segments, consisting of 232 segments from participants in the depressed (MDD) group and 248 segments from HC. The authors employed a train-test split strategy, where 80% of the data were used to train the model and 20% were held out exclusively for testing. This approach allows us to evaluate the model’s generalization performance on previously unseen data. The data were processed and loaded using custom-defined utilities built on top of PyTorch’s Dataset and DataLoader classes. Specifically, the train_test_datasets() function implemented the 80/20 split and returned corresponding dataloaders for both training and test sets.

The model’s performance was assessed using four key metrics: Accuracy (ACC), Precision (PRE), Recall (REC) and F1-Score. Accuracy quantifies the proportion of accurate predictions relative to the total number of samples. Precision is the accuracy of positive predictions, while Recall signifies the model’s sensitivity to true positives. The F1-Score, a harmonic mean of Precision and Recall, equilibrates these two measurements.

**Figure 5.**
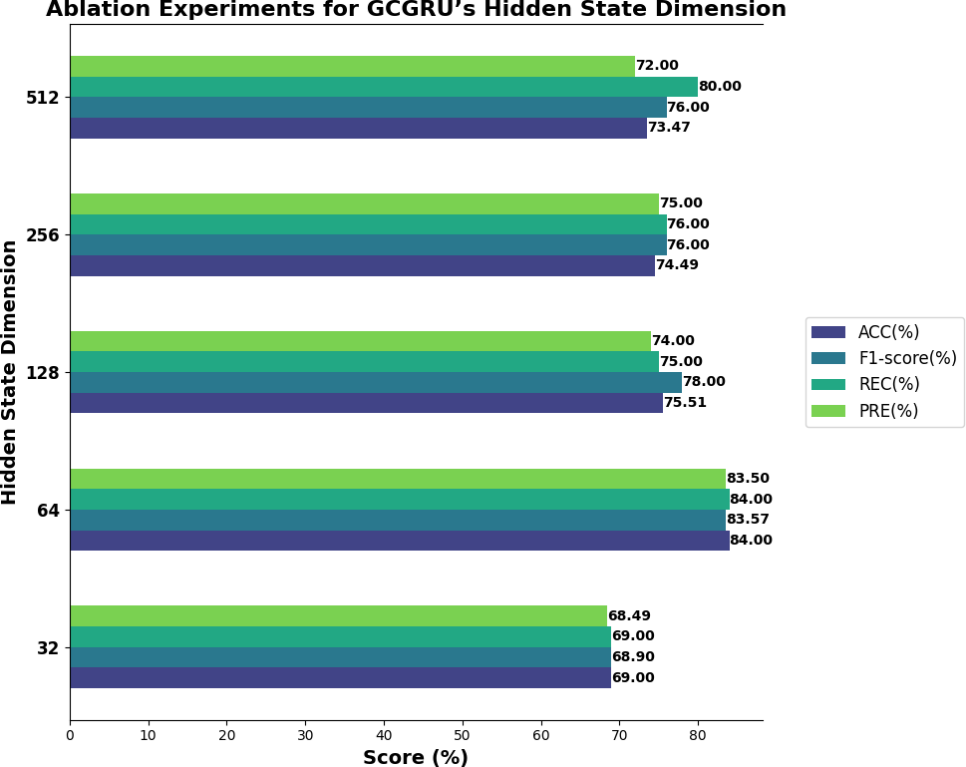
Performance of GCGRU on varying hidden states.

The GCGRU model is tested with varying hidden state dimensions (32, 64, 128, 256 and 512) as presented in figure 5, to identify the optimal feature representation. Observations indicate that lower dimensions, such as 32, limit the model’s ability to capture sufficient feature information, while larger dimensions, such as 512, introduce excessive parameters, risking overfitting. Ultimately, a hidden dimension of 64 was found to deliver the best balance between feature representation and model complexity, achieving optimal accuracy and F1-score.

### 4.2. Discussion

**Figure 6.**
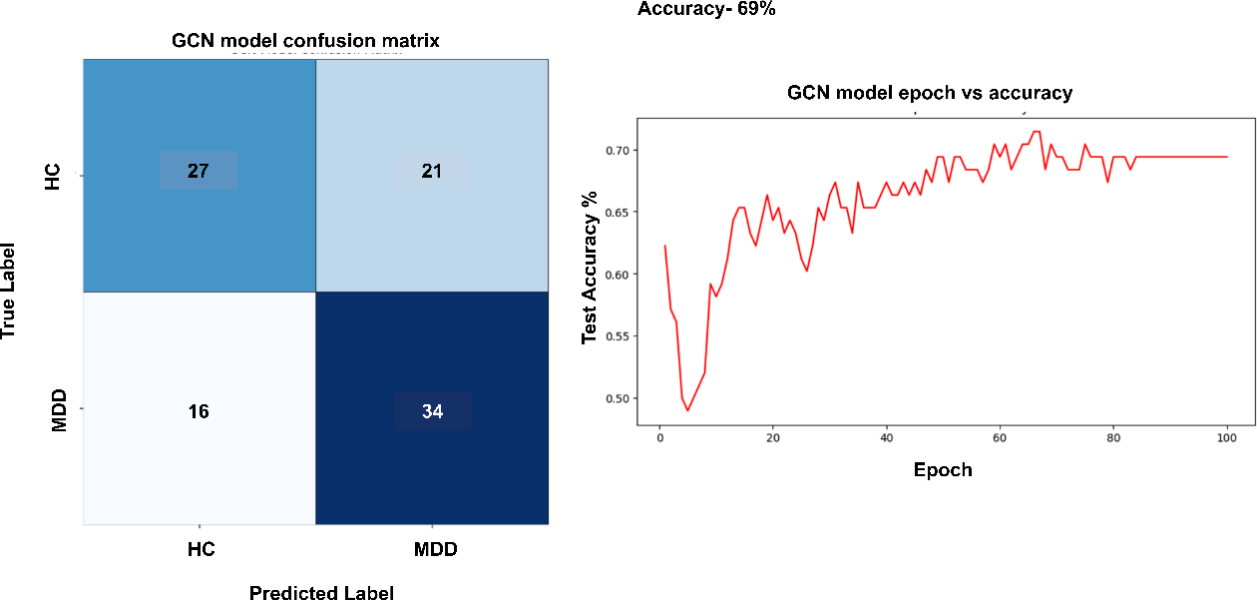
Performance of GCN module.

In this section, the proposed model is compared with its variants where the purposes of different components of the model are evaluated. First the improvement of using GCGRU instead of standalone GCN and GRU is given. Figure 6 shows the confusion matrix and the epoch frequency versus the testing accuracy curve for the standalone GCN module. The confusion matrix assesses the models' efficiency in predicting data into two classifications: HC and MDD. The epoch versus accuracy graph illustrates the optimal number of epochs required during model training to get the highest accuracy.

Figure 6 shows that the GCN model, which focuses on spatial feature extraction, has average classification performance. It accurately detects 27 HC samples and 34 MDD samples but incorrectly labels 21 HC samples as MDD, leading to a comparatively elevated false-positive rate. This suggests that while the GCN can identify spatial features in the EEG data, it has difficulties with class overlap which is overcome by incorporating temporal features. This results in an increased number of misclassified HC samples. From the epoch versus the testing accuracy curve, it can be seen that best accuracy is achieved at 100 epochs, so while training the model the number of epochs is set to 100 to get the best accuracy results.

**Figure 7.**
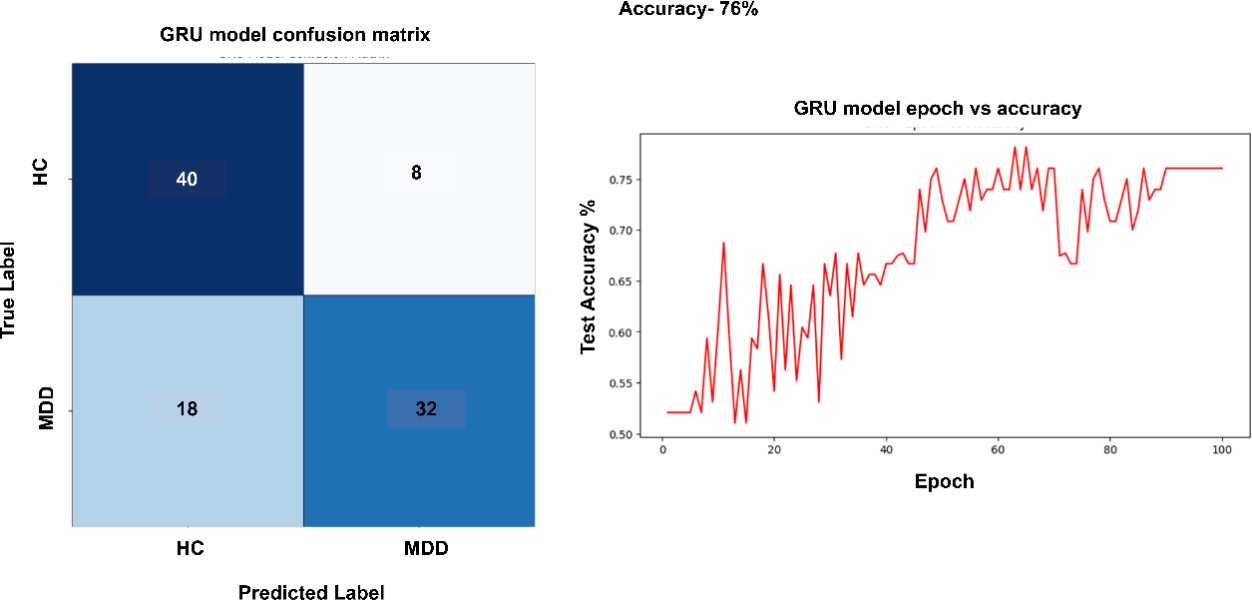
Performance of GRU module.

The GRU model shown in figure 7, which focuses on temporal correlations in EEG data, effectively identifies HC samples, yielding 40 true negatives and just 8 false positives. Nonetheless, it exhibits somewhat more difficulties with MDD samples, accurately identifying 32 while misclassifying 18 as HC, leading to an elevated false-negative rate for the MDD category. MDD impacts many areas of the brain. Consequently, the GRU’s emphasis on temporal factors, while neglecting spatial characteristics, leads to elevated false negative rates for MDD. Similarly, in GRU also, the number of epochs while training is set to 100.

**Figure 8.**
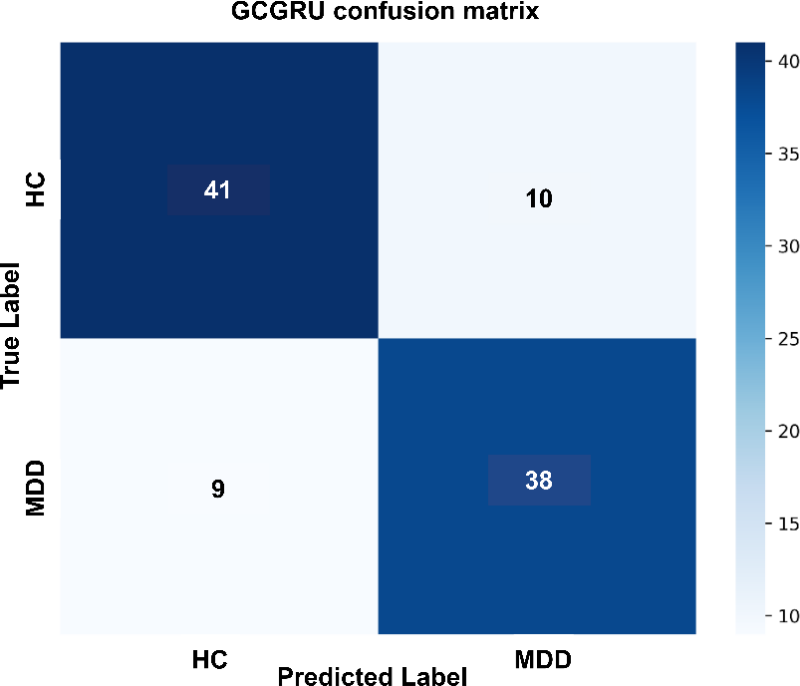
Performance of GCGRU model (proposed).

The combined GCGRU model, which integrates GCN’s spatial capabilities with GRU’s temporal processing, achieves the best overall performance. It correctly identifies 41 HC samples and 38 MDD samples, with only 10 HC samples misclassified as MDD and 9 MDD samples misclassified as HC. This balanced performance highlights the advantage of combining spatial and temporal feature extraction, as the GCGRU model reduces both false positives and false negatives, seen in figure 8, leading to more accurate classification for both cases.

**Figure 9.**
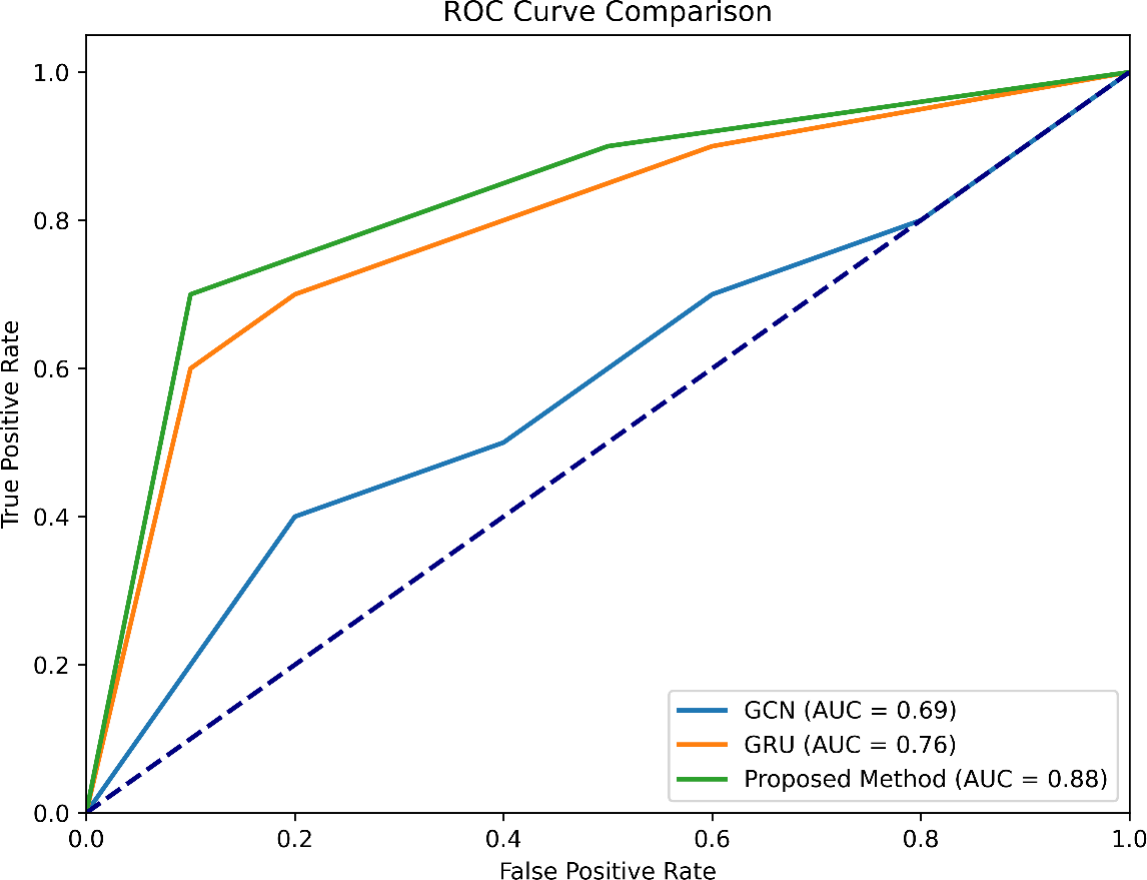
Comparative analysis of the ROC curve of proposed models, GCN and GRU.

**Figure 10.**
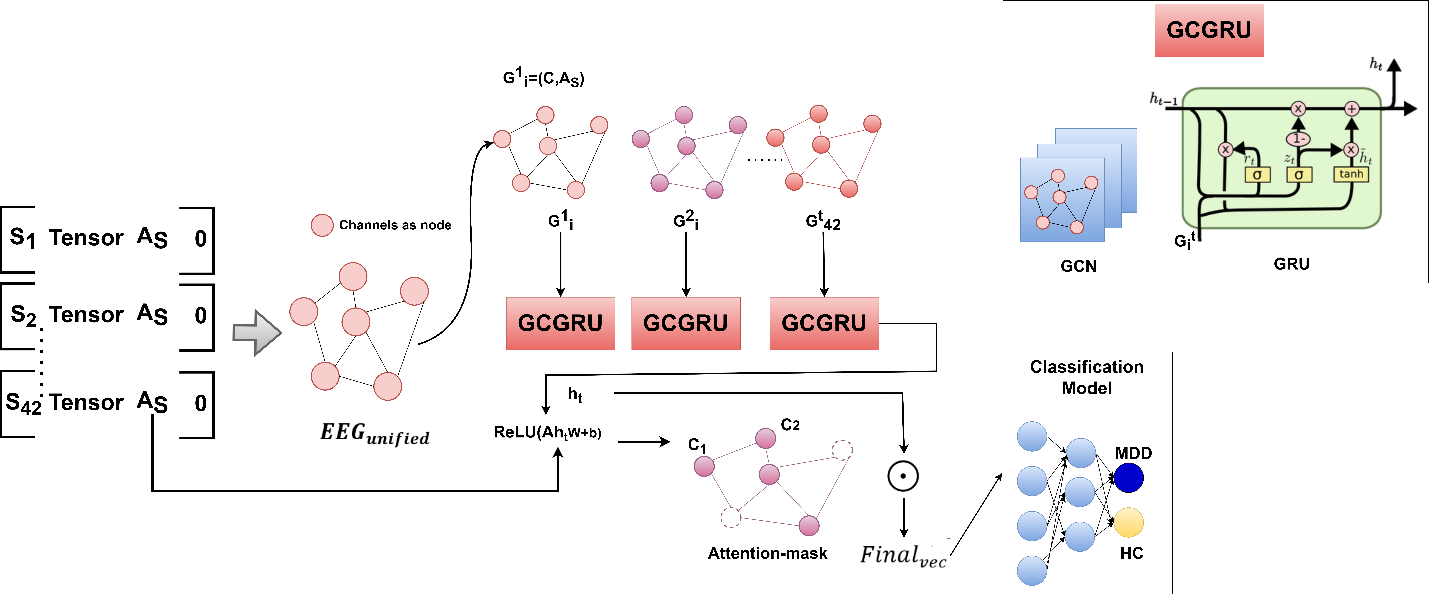
Illustration of process for depression detection using GCGRU model.

ROC-AUC (Area Under the Receiver Operating Characteristic Curve) is plotted for the proposed model, GCN and GRU model in figure 9. This is plotted to assess the trade-off between true positives and false positives. The proposed model shows the best performance among the three, with an AUC of 88%, which is 13.6 and 21.5% higher compared with the GRU and GCN models. This is because the model is able to correctly classify the MDD and HC labels with the help of KNN-based $A_{S}$ as shown in figures 2 and figure 10. The GRU model shows the value at 76% and GCN at 69%, respectively. With the implementation of max-pooling and creation of a unified brain network, the model is able to capture the high-order relations and provide high AUC.

Now the proposed model is compared with its variants to show the significance of each component. The variants that are used for comparison are (i) GCN: where only the GCN component of the proposed model is used and only captures spatial features of the data. (ii) GRU: where only the GRU component of the model is used capturing only temporal features of EEG data. (iii) $GCGRU_{N}$: is the proposed model without max-pooling and KNN- based adjacency matrix. (iv) $GCGRU_{MP}$: is the proposed model with max-pooling and without KNN- based adjacency matrix and (v) is the proposed model.

As seen from table 1, the proposed model has maximum performance compared to its variants. Although the variant that relies only on GCN to capture spatial dependencies across EEG channels efficiently learns spatial connections between various brain areas, it cannot pick up on time-dependent patterns in EEG data since it does not have any temporal context. Without temporal information, the model struggles to differentiate between classes based on time-sensitive EEG data, resulting in a baseline accuracy of 61%. The subsequent variation utilizes just the GRU component, emphasizing temporal relationships in EEG data. The sequential learning capability of GRU significantly enhances performance compared with GCN, since detecting depression requires understanding of the temporal dynamics of the EEG signal collected over time. Nonetheless, the absence of spatial information leads to the absence of critical relations across brain channels, resulting in marginally reduced accuracy. Although it lacks spatial information, there is a 12% performance increase over GCN.

This $GCGRU_{N}$ variation integrates GCN and GRU components, enabling it to collect both spatial and temporal data, hence enhancing the model’s comprehension of intricate EEG patterns with 76% accuracy. Integrating both feature types demonstrates a balanced enhancement compared to models that use just a single feature type. The integration of spatial and temporal variables improves performance by 3% compared with GRU alone and by 15% compared with GCN alone, highlighting the significance of incorporating both spatial and temporal features. The $GCGRU_{MP}$ model, enhanced with max-pooling, enables the capture of hierarchical patterns within the data, allowing the extraction of more significant features from the EEG channels. Graph max-pooling takes into account the topological information and node attributes of the unified brain network graph, effectively preserving the relevant information within the graph features. This allows the model to acquire more complex characteristics, hence improving its performance. The addition of max-pooling improves the accuracy by 3% over the $GCGRU_{N}$ variant, showing that hierarchical feature extraction contributes to a more robust performance.

The final variation, which is the proposed model, integrates both max-pooling in GCGRU and a KNN-based adjacency matrix $A_{S}$, allowing it to capture hierarchical patterns while improving spatial correlations across EEG channels. The KNN-based adjacency matrix ensures the representation of the most relevant spatial relationships among EEG channels, facilitating enhanced feature extraction accuracy. The KNN-based $A_{S}$ is designed to capture only the nearest and most spatially relevant connections between channels, as opposed to a Euclidean-based method that might create connections between every channel pair. Creating connections between every channel pair introduces irrelevant connections that are far away, which can reduce the significance of localized brain networks. Although in KNN-based $A_{S}$ *k* closest neighbours based on spatial proximity are considered, leading to reduced irrelevant information resulting in reduced noise. The proposed model has highest accuracy of 83.67% compared with the variants. The proposed model increases performance by approximately 5.67% over $GCGRU_{MP}$ and represents a 22.67% improvement over the baseline GCN model.

### 4.3. State-of-the-art comparison

In this section, the proposed model is compared with the state-of-the-art (SOTA) models used for depression detection using EEG signals in table 2. The SOTA models used are ShallowConvNet [19], DeepConvNet [19], EEGNet [20], TSception (Temporal Spatial Inception) [21], T-GCN (Temporal GCN) [22], DCGRU (Diffusion Convolutional Gated Recurrent Units) [23], LGGNet (Learning from local-global-graph) [24], AGTG (Adaptive Graph topology Generation model) [16], GCTNet [25], GCN [26] and GCN [27], which are widely cited in the EEG-based brain disorder classification field.

**Table 2.** Comparison of the proposed model with SOTA models.

model	ACC(%)	F1-score(%)	REC(%)	PRE(%)
ShallowConvNet [19]	68.76	77.95	92.82	68.21
DeepConvNet [19]	63.17	76.08	**99.22**	61.71
EEGNet [20]	65.62	77.03	97.38	63.82
TSception [21]	74.53	81.73	96.3	71.04
T-GCN [22]	65.87	77.06	97.1	63.87
DCGRU [23]	65.34	76.42	94.93	64.02
LGGNet [24]	77.7	82.39	88.47	77.22
AGTG [16]	77.78	82.75	90.23	76.46
GCTNet [25]	0.7693	77.66	87.88	77.89
GCN [26]	81.36	79.33	83.22	83
GCN [27]	83.17	83.9	83	83
proposed model	**83.67**	**84**	84	**84**

ShallowConvNet [19] and DeepConvNet [19] comprise convolutional layers alongside activation function for classification tasks. ShallowConvNet [19] consists of temporal and spatial relation extraction convolutional layers and then a squaring function, an average pooling layer, and a logarithm function. The ShallowConvNet [19] consists of only one convolutional layer and one pooling layer, while the DeepConvNet [19] consists of multiple convolutional and pooling layer blocks. Although these models capture spatial relations, they do not consider the temporal aspects and only use limit features to alpha, beta and gamma signals resulting in inferior accuracies. The proposed model has 21.67 and 32.47% more accuracy than ShallowConvNet [19] and DeepConvNet [19] models. EEGNet [20] leverages CNN with depthwise separable convolutions for feature extraction and demonstrates good robustness across various Brain Computer Interfaces (BCI) paradigms. Similar to ShallowConvNet [19] and DeepConvNet [19], EEGNet [20] also only uses spatial features and hence the accuracy for MDD classification is 21.58% lower than the proposed model. TSception [21], on the other hand, leverages both temporal and spatial features. In TSception, convolutional kernels are used to extract these features. As seen from the table 3, the TSception has better performance metrics compared with methods that only capture spatial relations. The T-GCN model [22], like the proposed model in this paper, considers both temporal and spatial dependencies. It generates graph-structured data based on geographical location and traffic speed, utilized for spatiotemporal traffic forecasting. This model uses graph-based structure for feature extraction and to capture topological information contained in the graph structure. This model serves as a good comparison benchmark for the proposed model. The DCGRU [23] uses graphs to capture the natural geometry of EEG sensors and dynamic brain connectivity. This model extends traditional RNNs with graph diffusion convolutions to model spatiotemporal dependencies in EEG signals using GRUs. This model effectively detects and classifies seizures. Although this model uses GRU and graphs to capture EEG features, it achieves inferior accuracy due to a lack of brain channel relation mappings. The LGGNet [24] model included previous neuroscientific information and delineated three categories of local–global graph structures, which were employed for feature fusion in the graph learning component based on local and global connections. But LGG-Net does not employ a hierarchical methodology, constraining its capacity to thoroughly investigate the spatial–temporal connections essential for EEG analysis. The proposed model has 7.13% increment in classification accuracy compared with LGGNet.

**Table 3.** Model performance with different components.

	ACC(%)	F1-score(%)	REC(%)	PRE(%)
GCN	61	61	61	61
GRU	73	74	73	73
$GCGRU_{N}$	76	76	76	76
$GCGRU_{MP}$	78	79	78	79
GCGRU model with max-pooling and KNN-based $A_{S}$ (**proposed**)	**83.67**	**84**	**84**	**84**

Similar to the model proposed in the current model, AGTG [16] utilizes graph-based feature extraction by the graph structure of the subjects and extracting the topological features from the graph-structured EEG data. A special graph max-pooling is also applied which selects the most prominent features after each convolutional layer. This model achieves closest accuracy to the proposed model in this paper. Utilizing a common unified brain network with spatiotemporal feature information for all the subjects alongside the KNN-enhanced adjacency matrix $A_{S}$ and GCGRU with graph max-pooling results in best accuracy among the SOTA models with an accuracy of 83.67% detection and, with F1-score, recall and precision reaching 84, 84 and 84%, respectively. The proposed model has 7.3% more accuracy compared with the AGTG [16] model.

Wand *et al.* developed a graph convolutional transformer network (GCTNet) to identify MDD accurately and reliably utilize EEG data. This system incorporates a Transformer block to extract global time-varying dynamics and a residual GCN block to collect spatial information. Furthermore, they provide the contrastive cross-entropy loss, which combines contrastive learning to improve classification performance by increasing the derived features’ stability and discriminability. The efficiency of the GCTNet model and CCE loss was evaluated using EEG data from 41 MDD subjects and 44 healthy controls, and a publicly accessible dataset. Using a subject-independent data partitioning strategy and 10-fold cross-validation, the suggested method achieved considerable performance, with average area under the curve values of 0.7693 and 0.9755 across both datasets [25].

Zhang *et al.* presented the GCN for classification of depression level. This approach addresses two challenges: (i) subjectivity in depression-level categorization owing to individual self-report biases and (ii) disparities in class across severity groups. This technique, inspired by model learning patterns, includes two unique modules: sample confidence and minority sample penalty. While the latter automatically upweights the incorrectly classified minority class data to solve imbalance difficulties, the former gradually filters EEG samples with weak label alignment during training by utilizing the L2-norm of pre-diction mistakes. This approach outperformed baseline models in multi-class severity recognition, achieving 81.13 and 81.36% accuracy across two public EEG datasets [26]. Further, Zhang *et al.* proposed a GNC-based (GNC) model that includes a secondary subject segmentation and attention mechanism. First, an attention module was provided that can focus on several channels with distinct characteristics at the same time. Second, a secondary subject partitioning technique is suggested to place subjects with comparable data patterns into the same domain with a common domain label, and domain extension based on adversarial training is incorporated into the model. This improves the domain generalization performance by efficiently decreasing the total quantity of domain labels and increasing the volume of data in each domain. Finally, the enhanced domain generalized concepts and attention modules work together to capture subject-invariant information in the depression recognition test. On two available datasets, prediction accuracy is 92.87 and 83.17%, respectively [27]. Another work used a reference subject-based validation technique to develope a handmade, computationally lightweight and self-organized classification model for accurate MDD identification. A new Twin Pascal’s Triangles Lattice Pattern with a 25-value array was developed for extracting local textural features from raw EEG signal. The obtained results showed 76.08% accuracy for Channel 1 and 83.96% accuracy for the top 13 channels for Multimodal Open Dataset for Mental Disorder Analysis [30]. Further, Tasci *et al.* developed two new conditional feature extraction methods that outperform existing local binary pattern (LBP) functions by using maximum and minimum distance vectors to produce patterns. The function's name was quantum LBP (QLBP). In addition, the wavelet packet decomposition was used to build a multilevel feature extraction algorithm. To choose the most useful features, the number of feature selection approaches was used, including ReliefF, Chi2, maximum relevance minimal redundancy and neighbourhood component analysis. After that, iterative hard majority voting was used to get the final predicted results. Using our multichannel electroencephalography real-time signal collection, channel-by-channel results were calculated and voted on to classify schizophrenia, intellectual disability and bipolar disorder. By using the k-nearest neighbours classification algorithm, the leave-one subject out cross-validation approach obtained maximum classification accuracy of 97.47, 94.36 and 93.49% for intellectual disability, schizophrenia and bipolar disorder, respectively. The comparison demonstrate the effectiveness of the proposed model's architecture in depression detection classification [31].

## 5. Conclusion

The article proposes a new DL framework with a unifed brain network with spatiotemporal features and a KNN-enhanced brain channel–channel relation matrix, aimed at improving the accuracy of depression detection by eliminating unnecessary EEG channels and effectively capturing complex relationships within brain networks. This framework integrates the analysis of channel–channel and subject–subject networks through a graph-based convolution and GRU temporal model, revealing intricate connections. The graph max-pooling helps the model to extract the intricate topological information from the graph structure and capture only important features. The performance of the proposed model is evaluated for the variants and with SOTA models. The proposed model shows 83.67% accuracy, 84% precision, 84% recall and 84% F1-score. The extensive discussion indicates that the proposed model effectively addresses the limitations of traditional methods by capturing hierarchical EEG data structures, resulting in enhanced accuracy and reliability in classification for practical applications. Future work will focus on improving brain network modeling techniques and continuing the investigation of variations in brain networks among different groups of individuals. The authors acknowledge the value of a dimensional approach, and future work may explore models predicting continuous BDI scores or subscale components to capture a more nuanced spectrum of depressive symptomatology. To strengthen the generalizability of our findings, the authors plan to extend this work in the future by increasing the number of subjects included in the dataset, which will allow for more robust, subject-level cross-validation and broader clinical application.

## Data Availability

The data that support the findings of this study are available at [32]. Supplementary material is available online [33].
